# Developing a robust and sensitive analytical method to detect off-flavor compounds in fish

**DOI:** 10.1007/s11356-022-19738-2

**Published:** 2022-03-23

**Authors:** Petra Camilla Lindholm-Lehto

**Affiliations:** Aquatic Production Systems, Natural Resources Institute Finland (Luke), Survontie 9A, FI-40500 Jyväskylä, Finland

**Keywords:** Depuration, Gas chromatography, Mass spectrometry, Method development, Off-flavors, Rainbow trout, Recirculating aquaculture system (RAS), Solid phase microextraction (SPME)

## Abstract

In recirculating aquaculture systems (RAS), off-flavors that accumulate in fish muscle tissue can be problematic in terms of consumer acceptance and the reputation of farmed fish products. Although off-flavors are not toxic at low concentrations, they often give fish muscle earthy, muddy, or other unwanted flavors. Traditionally, muddy off-flavors caused by geosmin (GSM) and 2-methylisoborneol (MIB) have been detected, but a variety of other compounds and flavors have also been identified. In this study, a method based on solid phase micro extraction (SPME) coupled with gas chromatography and mass spectroscopy was developed to identify and quantify 14 off-flavor–inducing compounds in RAS–farmed fish. The selected off-flavors were quantified in circulating water and in fish from a pilot-scale RAS rearing rainbow trout (*Oncorhynchus mykiss*). The method showed high accuracy and precision with limits of detection and quantification at a low ng L^−1^ level. In this study, 13 compounds were found in the fish muscle which decreased in concentrations during the 15-day depuration period. This study showed that off-flavors in fish can also be induced by other compounds besides GSM and MIB. This emphasizes the need for sufficient off-flavor control in the RAS, but also the importance of an accurate and reliable analytical quantitation method.

**Supplementary Information:**

The online version contains supplementary material available at 10.1007/s11356-022-19738-2.

## Introduction

Annual aquaculture production, including recirculating aquaculture systems (RAS), has reached 82.1 million metric tons (FAO 2018) and 46% of total fish consumption, and continues to increase. Unfortunately, off-flavors can be formed in the RAS due to microbial activity in aquaculture water and accumulate in fish muscle tissue. Off-flavors are typically produced as metabolic by-products of a variety of microbial species such as *Cyanobacteria* and *Actinobacteria* (Mahmoud and Magdy 2018), but the species *Myxobacteria* and *Sorangium* can also contribute to off-flavors and odors (Lukassen et al. 2017). Additionally, fish feed has also been suggested as a source of unwanted taste and flavors (Mahmoud et al. 2018). Lipid and protein compounds of feed ingredients can contain off-flavors while other compounds such as pyrazines can originate in thermal treatment in feed pellet formation (Mahmoud and Buettner 2017). Once formed, off-flavor compounds are relatively stable against chemical and biological degradation (Mahmoud and Magdy 2021).

Off-flavors perceived in fish are often described as musty and earthy flavors and odors that consumers find objectionable. These flavors are typically induced by the terpene compound geosmin (GSM, trans-1,10-dimethyl-trans-9-decalol) and 2-methylisoborneol (MIB, (1-R-exo)-1,2,7,7-tetramethyl-bicyclo[2.2.1]heptan-2-ol) (Gerber, 1968, 1969), although a wide variety of other compounds can cause unwanted flavors in fish muscle. These include a variety of alcohols, aldehydes, and terpenes (Selli et al. 2006; Podduturi et al. 2017; Mahmoud and Buettner 2017; Mahmoud et al. 2018). Moreover, the sensory threshold values for GSM and MIB are very low, and concentrations of 1.3–4.0 ng L^−1^ and 6.3–15 ng L^−1^ in water (Young et al. 1996; Watson 2004), and 250–900 ng kg^−1^ (GSM) and 700 ng kg^−1^ (MIB) in fish muscle, have been reported (Peterson et al. 1980; Grimm et al. 2004) and reviewed by Lindholm-Lehto and Vielma (2018). Although the detection of these very low concentrations can be difficult due to complex sample matrix of a RAS, it is possible with a suitable analytical equipment and method.

Freshly harvested fish have delicate but very distinctive flavors. The aromas in fish and other seafood can be classified as fresh fish-like flavors, species-related flavors, and flavors derived from processing and storage (oxidized, spoiled, putrid, and environment-derived flavors) (Lindsay 1990). The flavors of fresh fish and species-related flavors are typically highly appreciated by consumers and include 6-, 8-, and 9-carbon-chain aldehydes, ketones, and alcohols. Many of the compounds in fresh fish are also found in common vegetables (Tressl et al. 1982). During storage, some flavor compounds are transformed due to microbial interaction into less flavorful derivatives. Additionally, oxidation of lipids leads to flat, sweet, and putrid flavors. Microbes and, more specifically, bacteria are primarily responsible for the putrid flavors formed during the spoilage of fish (Reineccius 1979).

Methods to identify flavor-inducing compounds have been widely developed over the last decades (e.g., Mahmoud and Buettnet 2016; Ngai et al. 2019). Most utilize gas chromatography (GC), coupled with different detectors. However, even liquid chromatography coupled with an atmospheric pressure chemical ionization mass chromatography (LC-APCI) has been applied to quantify GSM and MIB in aquaculture water (Bedner and Saito 2020). Flavor compounds, as well as off-flavors, often occur in trace amounts and a sensitive analytical method is therefore required for their reliable detection and quantification. For example, Vrhovsek et al. (2014) developed a method based on GC–tandem mass spectrometry (MS/MS) for 160 volatile flavor compounds, including alcohols, ketones, aldehydes, and pyrazines in apples, grapes, and raspberries. Mahmoud and Buettner (2016) used high resolution GC coupled with olfactometry (HRGC-O) and split the gas flow between a flame ionization detector (FID) and a sniffing port for a team of experts to sniff the sample.

More recently, flavor-inducing compounds have been detected by a 1- and 2-dimensional GC–MS (Mahmoud and Buettnet 2016, 2017; Mahmoud et al. 2018). Mahmoud and Buettner (2016) used 2-dimensional HRGC-MS. The equipment consisted of two GCs with a DB-FFAP column for fatty acids and a DB-5 column of different polarity for the second dimension. The MS spectra were obtained in electron ionization (EI) mode and achieved a detection of 54 compounds, of which 47 were identified.

The detection and quantitation of off-flavor compounds are typically performed with a quadrupole MS or triple quadrupole (QQQ) MS in selected ion monitoring mode (SIM). A triple quadrupole (QQQ) MS in multiple reaction monitoring (MRM) mode is suitable for detecting low concentrations, despite the complex sample matrix which is often a feature of aquacultural samples. An ion trap mass spectrometer (Porcelli et al. 2021) is also an option, but SIM mode may be unavailable for the ion trap, and the SCAN mode does not allow sufficiently low detection limits (Parinet et al. 2011).

Before chromatographic separation, the extraction can be performed typically by purge and trap (Deng et al. 2012) or solid phase micro extraction (SPME, Petersen et al. 2011). SPME is an extraction method suitable for volatile and semi-volatile compounds such as the off-flavors GSM and MIB. Originally, it was developed by Pawliszyn and colleagues (Belardi and Pawliszyn 1989), combining sampling, extraction, and concentration without solvent addition. It can be conducted manually or by automation. An autosampler performs the laborious stages of the SPME: heating, sample stirring, penetration of the fiber into the sample vial, extraction, and injection into the GC inlet port in an automated and repeatable manner (Parinet et al. 2011). Unfortunately, there are disadvantages in SPME, such as expensive supplies, time-consuming conditioning of sorbents, and possible carry-over effects (Parinet et al. 2011; Cortada et al. 2011).

In our previous study (Lindholm-Lehto et al. 2019), we reported a method for analyzing GSM and MIB, the most commonly detected off-flavor compounds in aquaculture. To our knowledge, very few studies have been conducted to study other off-flavor compounds besides GSM and MIB. Selli et al. (2006) identified 38 compounds in rainbow trout by sensory and analytical identification but they did not quantify the compounds. Additionally, Mahmoud and Buettner (2017) detected odorant compounds in rainbow trout but they were not quantified. Only Podduturi et al. (2017) detected and semi-quantified volatile terpenes in pangasius (*Pangasianodon hypophthalmus*) and tilapia (*Oreochromis niloticus*). Overall, other compounds besides GSM and MIB have rarely been quantified. Here, we focused on the identification of several potential off-flavors and broadened our method for other off-flavor compounds besides GSM and MIB. The method was applied in studying the off-flavors in fish from a pilot-scale RAS and depuration. An accurate quantification can provide important information for producers aiming to improve their depuration procedure and produce fish of the highest quality.

## Materials and methods

### Chemicals

Reference compounds were purchased and applied for method validation and compound identification (Table 1) as follows: acetoin (3-hydroxy-butan-2-one, Sigma-Aldrich); hexanoic acid (Sigma-Aldrich/Supelco®); octanoic acid, hexanal, and octanal (Sigma-Aldrich); 2-methylisoborneol (MIB, 1-R-exo-1,2,7,7-tetramethyl-bicyclo[2.2.1]heptan-2-ol, 100 µg mL^−1^, TraceCERT®, Supelco®); 2-isobutyl-3-methoxypyrazine (IBMP, Sigma-Aldrich); 2-isopropyl-3-methoxy-pyrazine (IPMP, Tokyo Chemical Industry Co.); geosmin (GSM, trans-1,10-dimethyl-trans-9-decalol, 100 µg mL^−1^, TraceCERT®, Supelco®); 3-(methylthio)propionaldehyde, phenylacetaldehyde (PhenA), and α-terpineol (Alfa Aesar); 2,4,6-trichloroanisole (TCA); and vanillin (Sigma-Aldrich), NaCl solution (98%, Merck), methanol (MeOH ≥ 99.8%, J.T. Baker).

**Table 1:** Selected off-flavor compounds, their purities, formulas, CAS numbers, densities, and aroma descriptions

Compound	Purity	Chemical formula	CAS number	Density, g mL^−1^	Aroma
Acetoin /3-hydroxy-butan-2-one/	≥ 99%	CH_3_CH(OH)C(O)CH_3_	513–86-0	1.013	Buttery
Caproic acid/hexanoic acid	≥ 99%	CH_3_(CH_2_)_4_COOH	142–62-1	0.927	Goat-like
Caproic aldehyde/hexanal	≥ 95%	CH_3_(CH_2_)_4_CHO	66–25-1	0.815	Grass
Caprylic acid/octanoic acid	≥ 99.5%	CH_3_(CH_2_)_6_COOH	124–07-2	0.910	Fruity-acid, irritating
Caprylic aldehyde/octanal	99%	CH_3_(CH_2_)_6_CHO	124–13-0	0.821	Fruit-like
Geosmin/dimethyl-8-hydronaphtalen	99%	C_12_H_22_O	16,423–19-1	10^−4^	Musty
3-Isobutyl-2-methoxy- pyrazine	99%	C_9_H_1__4_N_2_O	24,683–00-9	0.990	Undesirable, musty
2-Isopropyl-3-methoxypyrazine	≥ 98%	C8H_12_N_2_O	25,773–40-4	0.996	Undesirable, musty
2-Methylisoborneol	99%	C_11_H_20_O	2371–42-8	0.968	Earthy
3-(Methylthio)propionaldehyde	98%	CH_3_S(CH_2_)_2_CHO	3268–49-3	1.052	Onion-like, meat-like
Phenylacetaldehyde	95%	C_8_H_8_O	122–78-1	1.075	Sweet, rose, flowery
α-Terpineol	96%	C_10_H_18_O	98–55-5	0.930	Terpenic
2,4,6-Trichloroanisole	99%	C_7_H_5_Cl_3_O	87–40-1	Solid	Medicinal, phenolic, iodine-like
Vanillin/4-hydroxy-3-methoxybenzaldehyde	99%	C_16_H_16_N_2_O_4_	1696–60-2	1.056	Vanilla, sweet

Besides GSM and MIB, off-flavors in RAS can be induced by different groups of compounds, such as aldehydes, alcohols, and terpenes (Podduturi et al. 2017). In this study, the compounds were selected based on feedback and descriptions received by a commercial RAS farm rearing rainbow trout, including consumer feedback and descriptions of professional cooks who tasted a batch of fish before depuration. Based on these descriptions, it was determined which compounds could induce the off-flavors and they were introduced to the analytical method.

### Manual assembly

Off-flavors were first extracted by manual headspace-solid phase microextraction (HS-SPME) before analysis. Aqueous sample (1 mL) or fish muscle tissue (1 g) was placed in a 10-mL HS vial with 750 µL of saturated NaCl solution. A volume of 30 µL of internal standard (IBMP, 90 ng L^−1^ in MeOH) was added, and the vial was closed with polytetrafluoroethylene (PTFE) septum caps (Merck).

The SPME was performed with a manual assembly as reported in Lindholm-Lehto et al. (2019). The analytes were adsorbed onto an extraction fiber coated with divinylbenzene/carboxene/polydimethyl siloxane (DVB/CAR/PDMS, 1 cm, 50/30 µm, part no. 57328-U) in a manual holder (Supelco®, Merck). The sealed vial was placed in a water bath at 60 °C. The septum of the sample vial was pierced with a needle, and the fiber was exposed in the headspace for 30 min. The fiber was then directly introduced into the GC injection port for desorption. The fiber remained in the inlet port for the entire runtime to allow full desorption and fiber conditioning between separate runs. The GC–MS equipment was controlled by MassHunter software, version B.05.01 (Agilent, Santa Clara, CA, USA).

### Autosampler

An autosampler assembly (CTC Analytics, Switzerland) was introduced to the GC–MS equipment to replace the more labor-intensive manual SPME procedure. The GC–MS was equipped with a PAL3 RSI 85 bundle with an 85-cm rail, D7/57 liquid tool, tray holder, and 3 VT54 racks, and alternatively, an arrow SPME tool (G7377A) and 3 VT15 racks. Additionally, the sampler included deck-mounting hardware (G7367A), a heatex stirrer (G7377A), a PAL3 SPME arrow kit for GC (G7377A 101), a conditioning module for PAL3 SPME (G7377A 201), and 3 sample racks for 10-mL vials (G7367A 402). Furthermore, an agitator incubation module (G7379A) and fittings for 10-mL vials (G6841AA) were included. For the SPME Arrow, a fiber made of DVB/carbon WR/PDMS was used, 1.10 mm in length and Ø120 µm, dark gray. The MassHunter software was updated to version B.10.0 to control the assembly.

The running conditions were optimized to control the PAL3 Arrow SPME autosampler. They included the selection of conditions for the time of sample mixing before adsorption, agitation speed, the time and temperature of adsorption, the time of desorption in the GC inlet port, and the time and temperature of fiber conditioning. The optimization was performed with an analysis method at GC-QQQ, aiming for optimal peak area of the analytes. Each new fiber was initially conditioned at 250 °C for 1 h prior to use.

### Analytical equipment

The volatile and semi-volatile compounds were desorbed using a GC and 7000 Series Triple Quadrupole mass spectrometer (GC-QQQ) (Agilent, Santa Clara, CA, USA). The EI/CI GC/MS interface included both a high sensitivity EI and CI ion sources, with the EI (70 eV) source connected (GC 7890A, detector interface G3440A, 7000 Triple Quadrupole MS/MS EI/CI bundle G7011AA).

A Phenomenex Zebron ZB-5MSi (Torrance, CA, USA) capillary column (30 m × 0.25 mm × 0.25 µm) was used to separate and detect the analytes. The injector was adjusted at 260 °C in the splitless mode, followed by the carrier gas (helium) at a flow rate of 1.2 mL min^−1^. The oven temperature started at 45 °C for 3 min increasing first 60 °C 18 °C min^−1^ and up to 300 °C 30 °C min^−1^ in 16.7 min. The GC/MS interface was operated at 280 °C.

The detection was performed in multiple reaction monitoring (MRM) mode. The conditions for each compound were determined as shown in Supplementary Table S1. The peak areas of the internal standard and analytes were used for quantification. Levels of detection (LOD) and quantification (LOQ) have been determined for each compound and listed for aqueous (Table 2) and for fish muscle (Table 3). No detectable concentrations of the analytes were found in the blanks.

**Table 2:** Limits of detection (LODs), quantification (LOQs), and linearities (*R*^2^) of the selected off-flavors in aqueous sample (1–100 ng L^−1^) analyzed with automated SPME-GC-QQQ

Compound	LOD	LOQ	Linearity, *R*^2^
Acetoin, ng L^−1^	0.45	0.80	0.9949
GSM, ng L^−1^	0.11	0.15	0.9861
Hexanal, ng L^−1^	0.73	1.23	0.9933
Hexanoic acid, ng L^−1^	1.61	5.50	0.9934
IBMP, ng L^−1^	1.26	1.26	0.9998
IPMP, ng L^−1^	0.12	0.53	0.9883
MIB, ng L^−1^	0.39	0.41	0.9918
Methional, ng L^−1^	1.04	1.40	0.9931
Octanal, ng L^−1^	0.29	0.96	0.9941
Octanoic acid, ng L^−1^	1.61	2.44	0.9949
Phenylacetaldehyde, ng L^−1^	0.32	0.78	0.9988
TCA, ng L^−1^	0.55	1.31	0.9959
α-Terpineol, ng L^−1^	0.85	1.05	0.9938
Vanillin, ng L^−1^	0.24	0.65	0.9800

**Table 3:** LODs, LOQs, and linearities (*R*^2^) of selected off-flavors in fish muscle (100–1000 ng kg^−1^) analyzed with automated SPME-GC-QQQ

Compound	LOD	LOQ	Linearity, *R*^2^
Acetoin, ng kg^−1^	41.1	45.7	0.9999
GSM, ng kg^−1^	23.0	65.1	0.9972
Hexanal, ng kg^−1^	35.4	99.4	0.9769
Hexanoic acid, ng kg^−1^	18.7	39.9	0.9999
IBMP, ng kg^−1^	29.8	99.6	0.9985
IPMP, ng kg^−1^	13.9	45.6	0.9999
MIB, ng kg^−1^	51.8	107.1	0.9988
Methional, ng kg^−1^	6.2	20.7	0.9984
Octanal, ng kg^−1^	30.2	100.7	0.9958
Octanoic acid, ng kg^−1^	28.3	94.4	0.9976
Phenylacetaldehyde, ng kg^−1^	16.0	20.2	0.9973
TCA, ng kg^−1^	27.3	91.0	0.9949
α-Terpineol, ng kg^−1^	13.4	51.3	0.9930
Vanillin, ng kg^−1^	14.0	46.6	0.9932

Cell energy values for each analyte were optimized and chosen using Agilent Optimizer software. Based on multiple runs and the peak areas of different conditions, the optimal cell energies were determined for each ionization (Supplementary Table S1).

### Experimental setup

The validated method was applied to study the flavor components in rainbow trout reared in a pilot-scale RAS. The full process description of the RAS and its water treatment procedures has been reported in Pulkkinen et al. (2021). In brief, the RAS (FREA Aquaculture solutions, Denmark) was located at the Laukaa fish farm of Natural Resources Institute Finland (Luke). The mean annual temperature in the area is 4 °C, and the annual precipitation is 600 mm (Finnish Meteorological Institute, 2020). The length of the growing season ranges from 165 to 175 days.

The RAS includes two identical units, each with two 5-m^3^ raceway fish tanks. The water flows through a 60-µm mesh-size drum filter (Hydrotech HDF800, Veolia, France), two parallel 2.5-m^3^ fixed bed bioreactors (each filled with 1.5 m^3^ Saddle-Chips, KSK Aqua, Denmark), a 2.24-m^2^ degassing unit, a 0.74-m^3^ pump sump, and finally a low-head oxygenator (FREA Aquaculture Solutions, Denmark) to the fish tanks. The pH of the circulating water was adjusted with NaHCO_3_ (Solvay Chemicals International SA, Brussels, Belgium). The temperature of the water remained at 16 °C, the pH at 7.0, and the oxygen in the rearing tanks at 8.0–10.0 mg L^−1^. On average, water was circulated via the water treatment field 8.5 m^3^ d^−1^ back to the RAS. Water from Lake Peurunka was used as clean replacement water if required.

The RAS effluent was treated in a passive water treatment field, consisting of a woodchip bioreactor (WB), constructed wetland, and a sand filter. The water flow was designed to follow a vertical flow via (14 m × 9 m × 1.5 m) WB with a hydraulic retention time of 48 h, aiming at denitrification for nitrate removal. The constructed wetland (7.5 m × 6 m) was designed for further N and P removal, but also for DOC removal, which may leach from the WB. Additionally, after the anaerobic denitrification stage, water was aerated at the CW. Finally, at sand infiltration, dissolved organic carbon and suspended solids were removed, as reported by Lindroos et al. (2002). A more detailed description of a passive water treatment field has been reported in Pulkkinen et al. (2021).

Water quality was monitored in the rearing tanks using online measurements, including carbon dioxide (Franatech, Germany), dissolved oxygen (Oxi:lyser, s::can, Austria), water flow rate (Fluxus F501, Flexim, Germany), water pH (ProMinent, Germany), and the inflow water rate (Watson Marlow 630, Spirax-Sarco Engineering, UK) from Lake Peurunka. The measurement data were stored on an industrial computer (Con::cube, S::can, Austria).

Additionally, the total ammonia nitrogen (TAN), nitrite, nitrate, and phosphate were analyzed on site using quick spectrophotometric tests (Procedure 8038 Nessler, LCK341/342, LCK340, and LCK349 respectively, DS 3900, Hach, USA). The water alkalinity was measured based on a standard titration method ISO 9963–1:1994 (TitraLab AT1000, Hach, Loveland, USA).

### Fish and feeding

Rainbow trout (*Oncorhynchus mykiss*), originating from the Hanka-Taimen facility, were reared in the experiment for 6 months. In this study, there were 1228–1535 fish, with an average weight of 1243–1437 g, a total biomass of 1764–1909 kg, and a tank density of 88–95 kg m^−3^. The fish were fed with a feed ratio of 1.20–1.25%, resulting in a feed conversion ratio (FCR) of 1.2. Supernumerary fish were regularly removed to maintain the tank biomass at a suitable level and the fish density below 100 kg m^−3^. The fish were fed by an automated feeding system (T Drum 2000, Arvo-Tec, Finland) with a commercial fish feed (BioMar Orbit, 6 mm) containing crude protein (37–40%), crude lipid (31–34%), carbohydrates (15.5–21.5%), ash (3.3–3.5%), and total phosphorous 0.8%, as given by the manufacturer. The fish were visually inspected on a daily basis.

In the summer, 100 fish were transferred from the system into a 48-m^3^ depuration tank. The depuration tank was operated in flow-through mode. The inlet water, a 1:1 mixture of surface water (depth of 3 m) and from the aphotic layer (depth of 8 m), was led from the oligotrophic Lake Peurunka (62.44886, 25.85201, area 694 ha, 59 600 m^3^) at a rate of 8 L s^−1^ (700 m^3^ d^−1^). Feed was withheld during depuration.

The fish were sampled from the rearing tank and a depuration tank after 1, 5, 7, 11, 13, and 15 days of depuration. In each sampling, three fish were selected and humanely euthanized. The fish were weighed, gutted, filleted, and sampled from the lateral part of fillet as reported by Hathurusingha and Davey (2016). They were stored at − 24 °C before the analysis. Pooled samples of the three individuals were used for the off-flavor analyses.

The study followed the protocols approved by the Luke Animal Care Committee, Helsinki, Finland, and EU Directive 2010/63/EU for animal experiments.

### Statistical analyses

Statistical analyses were performed with IBM SPSS Statistics for Windows, Version 26.0 (Armonk, NY: IBM Corporation, released 2019). Linear regression analysis was used to calculate the *R*^2^, and standard error to calculate LODs and LOQs, and evaluate the linearity. The confidence interval was set at 95%.

## Results and discussion

### Method validation

#### Linearity

The first aim of this study was to validate the developed method for both aqueous and solid fish samples. The concentrations in RAS water are often very low (low ng L^−1^ range) and the validation was performed with five points at concentration range of 0–100 ng L^−1^ for each compound. For all the selected off-flavor compounds, the linearities were close to 1 (Tables 2 and 3).

#### Limit of detection (LOD), limit of quantification (LOQ)

The limits of detection and quantification were determined (Tables 2 and 3) and the signal to noise (S/N) ratio was determined for each compound (3S/N for LOD and 10S/N for LOQ). According to these results, the method was suitable for quantifying the compounds at low concentrations.

#### Accuracy

It is considered that precision and accuracy are good when the error remains below 5%. Accuracy of the method, including SPME, was evaluated by running standard samples with known concentrations of the low and high ends of the analytical range. The results have been listed in Supplementary Table S2. The results were in good agreement, ranging from 96 to 104%, leaving the error below 5%.

#### Precision

Precision was evaluated by making repeated analyses of similar standard samples. Multiple samples were run (*n* = 5) per day and during the 3 following days. Based on them, the day-to-day precision and intraday precision were determined for each compound as reported in Destandau et al. (2005). The results of intraday and day-to day precision for aqueous samples have been listed in Supplementary Table S3.

Overall, the method showed low LODs and LOQs for each compound with good linearities. In comparison, Culleré et al. (2011) reported a method based on SPE GC–MS with good linearities for aldehydes, including octanal but the LODs remained at 3–40 ng L^−1^. For the detection requirements of the RAS and the demand of very low LODs, these levels may be too high.

Lian et al. (2021) quantified only GSM and MIB. They reached LOD of 0.6 µg kg^−1^ and LOQ 1.0 µg kg^−1^ for both 2-MIB and GSM in fish. In contrast with their report, 1.0 µg kg^−1^ concentrations can be observed by the human senses, suggesting there is room for improvement in the analytical method.

In addition, Dennenlöhr et al. (2020) reached quantification in the 0.01–1000-µg L^−1^ range for the analysis of aldehydes in wort and beer by HS-SPME GC-EI-MS/MS. Podduturi et al. (2021) used stir bar sorptive extraction (Twister®) with GC–MS analysis for depuration of water in a RAS, and reached LODs and LOQs below 1 ng L^−1^ for both GSM and MIB. For solid fish samples, they used dynamic headspace analysis and reached LOQs of 100 ng kg^−1^ for GSM and MIB (Podduturi et al. 2017, 2021). They are in a similar quantification range compared to results of this study.

### Comparison of manual and autosampler SPME

The method validation was first performed with manual SPME. The LODs and LOQs were determined and listed in Supplementary Table S4. The results showed that although the analytical procedure with the chromatographic separation and detection was unchanged, the more advanced and repeatable SPME procedure with a PAL3 sampler led to a more precise procedure with lower LODs and LOQs. Although the same fiber material was used, it was a little longer (110 mm vs. 100 mm) and thicker (120 µm vs. 50/30 µm) than that in the manual holder, creating a larger area for the analyte adsorption. Furthermore, the PAL3 sampler was equipped with a Heatex stirrer (G7377A), allowing better stabilization of the analyte in the headspace before the adsorption. All this explains the improved validation results (Tables 2 and 3, Supplementary Table S4).

The results of lower LODs and LOQs with automated SPME are in agreement with previous studies. For example, Wert et al. (2014) used automated SPME and analyzed with a GC–MS/MS in EI mode, similar to our study. They achieved LOQs of 0.596 ng L^−1^ (MIB) and 0.589 ng L^−1^ (GSM) with recoveries ranging from 88 to 110%. Although Wert et al. (2014) studied fewer compounds, the method LOQs and recoveries were in a similar range to the results of this study. On the other hand, Podduturi et al. (2020) used stir bar sorptive extraction (SBSE, Twister®) coupled with GC–MS. They achieved good reproducibility (RSD < 3%), linearity (*R*^2^ = 0.99), and LODs of 1 ng L^−1^ for both GSM and MIB. However, the method was not applied for solid (fish muscle) samples.

In our previous study (Lindholm-Lehto et al. 2019), we examined different SPME fiber materials, polyacrylate (PA), PDMS, PDMS/CAR, and DVB/CAR/PDMS, finding the best sensitivity with DVB/CAR/PDMS. In many previous studies, the DVB/CAR/PDMS material has shown the best results in SPME (Godelman et al. 2008; Kotseridis et al. 2008; Parinet et al. 2011; Botezatu et al. 2014). On the other hand, Hjelmeland et al. (2016) studied five different fiber materials for SPME (PDMS, PA, DVB/CAR/PDMS, CAR/PDMS, and DVB/PDMS), finding that DVB/PDMS and DVB/CAR/PDMS gave the highest peak areas, but the latter with carryover, and DVB/PDMS was therefore chosen. This suggests that the fiber material should be chosen based on the set of analytes, and the analysis conditions should be optimized.

### Off-flavors in the RAS and in depuration

The concentrations of the selected off-flavor compounds ranged from below LOD (e.g., TCA) to 124 ng L^−1^ (α-terpineol) in the circulating water (Table 4). Among the 14 selected off-flavor compounds, 8 were found in the rearing tank water. The results of water quality measurements in the rearing tank have been listed in Supplementary Table S5. In the inlet water from Lake Peurunka, the concentrations were in many cases below the LODs, but 77 ng L^−1^ of α-terpineol, 2.7 ng L^−1^ of GSM, 8.3 ng L^−1^ of MIB, and 69 ng L^−1^ of octanal were found (Table 4). Concentrations lower than 15 ng L^−1^ GSM and MIB in water can induce off-flavors in fish (Howgate 2004). Unfortunately, similar limit values have not yet been determined for the other compounds.

**Table 4:** Concentrations of the selected off-flavors detected in the rearing tank water of the RAS and in the inlet water from Lake Peurunka (ng L^−1^, ± SD, *n* = 4)

Compound	Rearing tank	Inlet water
Acetoin, ng L^−1^	< LOD	< LOD
GSM, ng L^−1^	15.4 ± 0.7	2.7 ± 0.1
Hexanal, ng L^−1^	< LOD	< LOD
Hexanoic acid, ng L^−1^	6.2 ± 0.3	5.4 ± 0.3
IBMP, ng L^−1^	< LOD	< LOD
IPMP, ng L^−1^	< LOD	< LOD
MIB, ng L^−1^	74.9 ± 3.7	8.3 ± 1.0
Methional, ng L^−1^	62.1 ± 2.9	41.8 ± 1.7
Octanal, ng L^−1^	92.4 ± 4.1	10.3 ± 1.1
Octanoic acid, ng L^−1^	< LOD	< LOD
Phenylacetaldehyde, ng L^−1^	8.9 ± 0.4	< LOD
TCA, ng L^−1^	< LOD	< LOD
α-Terpineol, ng L^−1^	124.3 ± 5.1	77.1 ± 3.6
Vanillin, ng L^−1^	9.3 ± 1.5	< LOD

< LOD below the limit of detection.

Small concentrations of GSM and MIB are typically found in surface water during the summer and produced, for example, by Cyanobacteria (Wang et al. 2015). Limonene (1-methyl-4-(1-methylethenyl) cyclohexene) occurs naturally in certain trees and bushes, including conifers (Strömvall 1992). Limonene can be biotransformed to α-terpineol by certain fungal strains (*Penicillium digitatum*, Tai et al. 2015), which may explain the relatively high concentration of α-terpineol in the inlet water. Octanal, hexanal, and methional occur in nature, e.g., in certain berries (Wang et al. 2005). Additionally, blooms of certain algal groups (*Chrysophyceae, Cryptophyceae, Dinophyceae, Chlorophyceae*) can produce compounds with characteristic odors, including alkenes, aliphatic alcohols, aldehydes, ketones, esters, thioesters, and sulfides (Jüttner 1983), and be the source of these compounds in the inlet water.

**Fig. 1:**
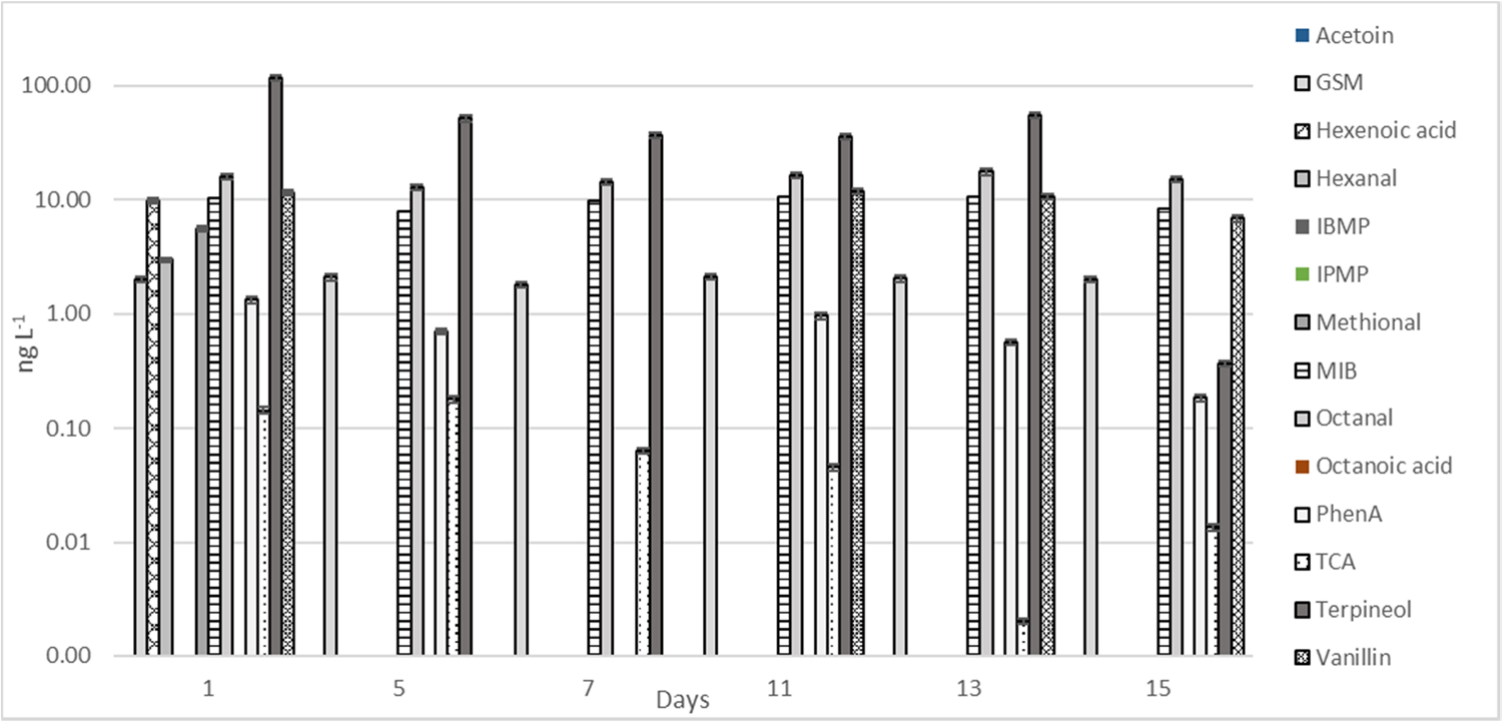
Concentrations of 14 off-flavor compounds in the depuration tank water, days 1–15 (ng L^−1^, ± SD, *n* = 4). Abbreviations: GSM geosmin, IBMP 3-isobutyl-2-methoxypyrazine, IPMP 3-isopropyl-2-methoxypyrazine, MIB 2-methylisoborneol, PhenA phenylacetic acid, and TCA 2,4,6-trichloroanisole. Acetoin, IBMP, IPMP, and octanoic acid were not detected (< LOD)

In depuration, the highest number of compounds and highest concentrations were found in the beginning of the period (Fig. 1). For most of the compounds, the concentrations decreased during the 15 days. However, concentrations of octanal remained at a constant level (12–17 ng L^−1^) throughout depuration. Hexenoic acid, methional, hexanal, and phenylacetic acid were found on day 1, but they decreased rapidly to below 1 ng L^−1^ (Fig. 1, Supplementary Table S6). Low concentrations of GSM (1.8–2.1 ng L^−1^) and MIB (8–11 ng L^−1^) were found even at the end of depuration, but this is probably explained by the concentrations found in the inlet water (Table 4).

There was a slight increase in concentrations of MIB, terpineol, and vanillin after 11 days of depuration (Fig. 1, Supplementary Table S6). This could be caused by the variations in inlet water from Lake Peurunka. The depuration period was run in flow-through mode, and the water was led directly into the depuration tank. The results show therefore a good representation of the inlet water.

**Fig. 2:**
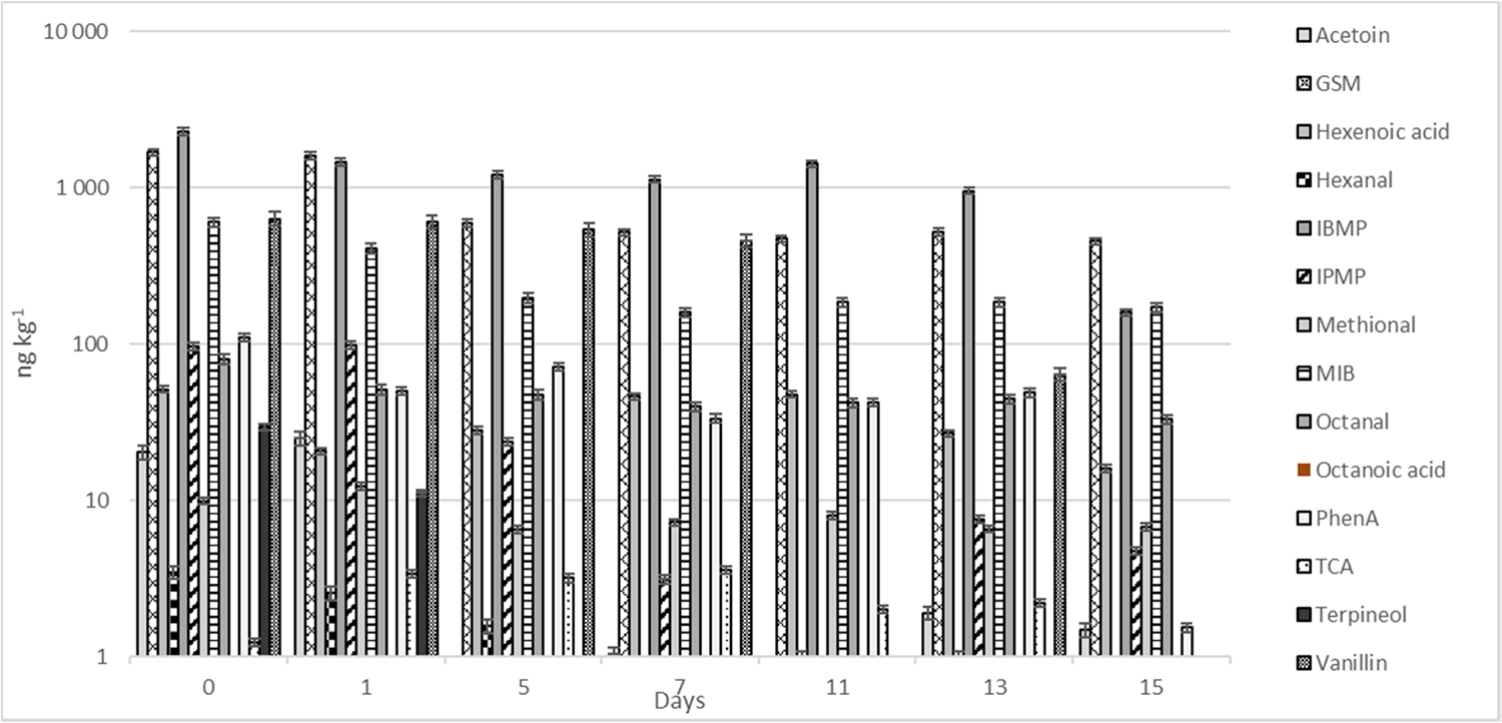
Concentrations of 14 off-flavor compounds in fish muscle in the rearing tank of the RAS (day 0) and during the depuration period, days 1–15 (ng kg^−1^, ± SD, *n* = 4). Abbreviations: GSM geosmin, IBMP 3-isobutyl-2-methoxypyrazine, IPMP 3-isopropyl-2-methoxypyrazine, MIB 2-methylisoborneol, PhenA phenylacetic acid, and TCA 2,4,6-trichloroanisole. Octanoic acid was not detected (< LOD)

The highest concentrations of the off-flavor compounds in fish muscle were found in the rearing tank (Fig. 2, Supplementary Table S7). In the rearing tank, the concentrations of GSM peaked at 1.7 µg kg^−1^, IBMP 2.3 µg kg^−1^, and 600 ng kg^−1^ for MIB, while the others remained below the 150-ng kg^−1^ level. The concentrations of GSM and MIB can be considered typical compared to those previously reported for GSM, 270–590 ng kg^−1^ and MIB 4.8–19.7 µg kg^−1^ (Zimba et al. 2012), 6 µg kg^−1^ GSM (Sarker et al. 2014), and 100–350 ng kg^−1^ GSM (Schrader et al. 2013) in rainbow trout before depuration. All the detected concentrations decreased during depuration, including GSM to 520 ng kg^−1^, IBMP to 160 ng kg^−1^, and MIB to 170 ng kg^−1^.

Besides GSM and MIB, other off-flavor compounds in fish muscle have been studied only rarely. Podduturi et al. (2017) studied and semi-quantified some terpene compounds and found α-terpineol at 10–400 ng kg^−1^ in pangasius and 50–1500 ng kg^−1^ in tilapia, while we found only up to 30 ng kg^−1^ in rainbow trout and up to 124 ng L^−1^ in water. The odor threshold for α-terpineol in water ranges from 330 to 350 µg L^−1^ and is therefore an unlikely cause of off-flavor or odor in this study.

The concentrations of GSM and MIB remained below 15 ng L^−1^ in the inlet water (and depuration) which is considered the sensory threshold value for GSM and MIB in water (Young et al. 1996; Persson 1980; Howgate 2004). The low concentrations in water led to decreased levels during depuration although some GSM (520 ng kg^−1^) was found in fish muscle even after 15 days (Figs. 1 and 2). However, they remained below the sensory threshold in fish (0.1–0.7 ng g^−1^ MIB, 0.25–0.9 ng g^−1^, Grimm et al. 2004; Robertson et al. 2005). Like the results in water (Fig. 1, Supplementary Table S6), there was a slight increase in certain compounds (MIB, octanal, PhenA, and IBMP) after 11 days of depuration (Fig. 2, Supplementary Table S7).

Even in the basic handbooks of aquaculture and RAS, farmers are instructed to taste or analyze off-flavors from each fish batch before selling to customers (Timmons et al. 2018). However, there are differences between persons in their abilities to sense low levels of off-flavors (Howgate 2004) and tasting a high number of different compounds can be tricky. Therefore, a reliable and accurate analytical quantification method can be valuable, such as the method presented in this study.

The concentrations of IBMP were relatively high (159–2282 ng kg^−1^) in fish muscle throughout the experiment (Fig. 2), especially in the rearing tank (2282 ng kg^−1^, Fig. 2), although the concentrations in water remained below LOD. IBMP may originate in the feed, and more precisely from the thermal treatment during feed pellet production, as suggested by Mahmoud and Buettner (2017) and Mahmoud et al. (2018). In this case, IBMP would accumulate in the fish muscle via the feed and intestinal tract, not via water. The fish were not fed during depuration, and the concentration of IBMP decreased during depuration (Fig. 2).

In addition to the content of the inlet water and microbial products in nature, as well as in the RAS, fish feed can induce some off-flavor compounds in the system. The main groups of ingredients are lipids and proteins in fish feed (Shen et al. 2018), and certain volatile aldehydes, for example, can originate in the lipid sources (Turchini et al. 2007). Additionally, the replacement of fish oil with pig lard can increase the risk of accumulation of fecal-like (skatole) and sweat-like (androstenone) taste and odors in fish muscle (Zhou et al. 2015), although such an ingredient is not used in salmon and trout feeds.

Sensory threshold values in fish muscle have not been defined for many of the selected off-flavor compounds. However, some perspective can be obtained by the threshold values found in the literature, although it is often determined for other sample matrices. Culleré et al. (2011) studied aldehydes in wines and reported an odor threshold for octanal of 1.75 µg L^−1^ in wine. Octanal is a compound with a powerful aroma and has a sensory threshold of 0.7 µg L^−1^ in water (Culleré et al. 2011). Da Costa et al. (2004) reported sensory threshold values of lower than 1.0 µg L^−1^ for phenylacetaldehyde, 0.5 µg L^−1^ for beer, and 0.2 µg L^−1^ in water for methional (Wang et al. 2005). Although the threshold values were evaluated in matrices other than fish muscle, this may imply that the concentrations of low ng L^−1^ level also remain below the sensory threshold values in fish.

A somewhat higher sensory threshold was reported for hexanoic acid at 1.8–3.6 mg L^−1^ and for octanoic acid, 0.16–1.9 mg L^−1^, in tea (Van Gemert 2003; Zhu et al. 2017). Furthermore, acetoin has a relatively high sensory threshold of 150 mg L^−1^ in wine (Ehsani et al. 2009). In this study, its concentrations were very low (16–51 ng kg^−1^ for hexanoic acid, below LOD for octanoic acid, 1.0–25 ng kg^−1^ in fish, and below LOD in water for acetoin). Although the matrices reported in the literature differ from those of this experiment (water, fish muscle), the concentrations remain lower than the sensory thresholds reported in the literature, and it is unlikely that they lead to an off-flavor sensation in fish.

Much lower sensory threshold values were reported for methoxypyrazines, including IPMP at 2 ng L^−1^ and IBMP at 1–2 ng L^−1^ in wine (Sala et al. 2004; Godelmann et al. 2007) and in water (IPMP and IBMP 2 ng L^−1^, Li et al. 2016), which emphasizes the need for very low method LODs and LOQs. Although the levels remained below LOD in water, up to 2.3 µg kg^−1^ IBMP was found in fish muscle. The compounds may originate in the thermal treatment in feed pellet formation (Mahmoud and Buettner 2017) and the decreased in depuration, because the feed was withheld during depuration.

The K_ow_ of TCA is 3.91, and solubility in water only 10 mg L^−1^ (Li et al. 2016), which suggests a greater tendency to accumulate in the fish muscle. The sensory threshold value for TCA is very low (1–10 ng L^−1^ in water, Cravero et al. 2015; 7 ng L^−1^ in water Li et al. 2016). TCA is a methylation product of trichlorophenols and is produced by certain microbial strains (*Penicillium, Aspergillus, Actinomyces, and Streptomyces*), many of which can also produce GSM and MIB (Lukassen et al. 2017; Mahmoud and Magdy 2018). However, the concentrations of TCA remained below the LOD in water and in fish, inducing a negligible risk of off-flavor sensation.

## Conclusions

The aim of this study was to identify and quantify potential off-flavors in RAS and in depuration. An analytical method was developed for this purpose based on automated SPME followed by GC-(EI) MS/MS analysis and developed for the detection of 14 selected off-flavors which could induce off-flavors in fish. The developed method was able to quantify the compounds with good linearity and low LODs and LOQs with high repeatability and precision. The method was applied for aqueous and fish muscle samples from a RAS and a depuration procedure of 15 days. In addition to GSM and MIB, the results showed several compounds inducing unwanted taste and flavor. Of 14 compounds, 13 were identified in fish, the lowest at 0.8 ng kg^−1^ (hexanal), the highest at 2.3 µg kg^−1^ (IBMP). All compounds decreased in concentration during depuration, but 160 ng kg^−1^ of IBMP and 520 ng kg^−1^ of GSM were still found after 15 days.

In conclusion, several other off-flavor compounds, additional to GSM and MIB, were quantified in RAS when aiming to produce good quality fish. Based on comparisons with values found in the literature, the detected compounds decreased to below the sensory threshold values in fish muscle and were unlikely to cause unwanted off-flavor after depuration. The method of this study increases possibilities to monitor the behavior of the off-flavor compounds in RAS and enables increasing the understanding of the off-flavor accumulation.

Supplementary information.

## Supplementary Information

Below is the link to the electronic supplementary material.

Supplementary file1 (DOCX 57 KB)

## Data Availability

The datasets used and/or analyzed during the current study are available from the corresponding author on reasonable request.
