# Supplementary material for: Developing a robust and sensitive analytical method to detect off-flavor compounds in fish
Source: Environ Sci Pollut Res Int. 2022 Mar 23;29(37):55866–76. doi: 10.1007/s11356-022-19738-2 (PMC9374641; doi:10.1007/s11356-022-19738-2)
Supplement: Supplementary file 1 — Supplementary file1 (DOCX 57 KB) [file 11356_2022_19738_MOESM1_ESM.docx]

Supplementary Table S1. Selected parent ions, *m/z*, daughter ions *m/z*, dwell times, ms and cell energies, eV determined for detection of analytes.

| **Compound** | **Parent ion, *m/z*** | **Daughter ion, *m/z*** | **Dwell, ms** | **CE, eV** |
| --- | --- | --- | --- | --- |
| Acetoin | 73.2 | 59 | 150 | 20 |
| Acetoin2 | 72.8 | 59 | 150 | 20 |
| GSM1 | 96.9 | 67 | 150 | 15 |
| GSM2 | 111.9 | 97 | 150 | 10 |
| GSM3 | 126 | 82.9 | 150 | 20 |
| Hexanal1 | 100.0 | 82.1 | 150 | 10 |
| Hexanal2 | 100.0 | 72.1 | 150 | 0 |
| Hexanal3 | 82.0 | 67.1 | 150 | 5 |
| IPMP1 | 153.3 | 158.2 | 150 | 10 |
| IPMP2 | 124.3 | 119.1 | 150 | 20 |
| MIB1 | 94.9 | 69 | 150 | 5 |
| MIB2 | 95 | 67 | 150 | 10 |
| MIB3 | 107 | 69 | 150 | 15 |
| Octanal1 | 100.1 | 95.1 | 150 | 35 |
| Octanal2 | 82.2 | 69.2 | 150 | 20 |
| Octanal3 | 82.2 | 57.2 | 150 | 0 |
| Octanoic acid1 | 115.1 | 87.2 | 150 | 5 |
| Octanoic acid2 | 101.2 | 72.8 | 150 | 5 |
| PhenA1 | 120.3 | 92.3 | 150 | 0 |
| PhenA2 | 120.2 | 65.2 | 150 | 25 |
| PhenA3 | 92.2 | 63.1 | 150 | 25 |
| Terpineol1 | 135.8 | 107.1 | 150 | 5 |
| Terpineol2 | 120.8 | 95.2 | 150 | 15 |
| TCA1 | 212.0 | 197.1 | 150 | 15 |
| TCA2 | 209.9 | 194.9 | 150 | 15 |
| TCA3 | 166.9 | 108.9 | 150 | 20 |
| Vanillin1 | 150.8 | 123 | 150 | 5 |
| Vanillin2 | 137.0 | 109 | 150 | 10 |

Supplementary Table S2. Accuracy (%) of spiked and measured off-flavor compounds at low‑end and high‑end concentrations (ng L^-1^) in the linear range of the method.

| **Compound** | **Spiked, low end** | **Measured, low end** | **Recovery (%)** | **Spiked, high end** | **Measured, high end** | **Recovery (%)** |
| --- | --- | --- | --- | --- | --- | --- |
| Acetoin, ng L^-1^ | 5.00 | 5.02 | 100.3 | 150.0 | 152.4 | 101.6 |
| GSM, ng L^-1^ | 3.00 | 3.10 | 103.4 | 150.0 | 149.1 | 99.4 |
| Hexanal, ng L^-1^ | 3.86 | 3.71 | 96.1 | 150.0 | 150.0 | 100.0 |
| Hexanoic acid, ng L^-1^ | 10.0 | 10.7 | 100.7 | 100.0 | 104.7 | 104.7 |
| IBMP, ng L^-1^ | 3.00 | 2.94 | 98.0 | 100.0 | 99.1 | 99.1 |
| IPMP, ng L^-1^ | 5.00 | 5.23 | 104.6 | 150.0 | 149.7 | 99.8 |
| MIB, ng L^-1^ | 3.00 | 3.03 | 100.9 | 150.0 | 147.9 | 98.6 |
| Methional, ng L^-1^ | 5.00 | 4.94 | 98.7 | 100.0 | 101.7 | 101.7 |
| Octanal, ng L^-1^ | 3.28 | 3.42 | 104.3 | 130.0 | 128.6 | 98.9 |
| Octanoic acid, ng L^-1^ | 5.00 | 4.975 | 99.5 | 150.0 | 153.2 | 102.1 |
| Phenylacetaldehyde, ng L^-1^ | 5.00 | 5.12 | 102.4 | 50.0 | 50.5 | 101.0 |
| TCA, ng L^-1^ | 5.45 | 5.43 | 99.6 | 145.0 | 143.9 | 99.2 |
| Terpineol, ng L^-1^ | 3.36 | 3.33 | 99.1 | 150.0 | 148.7 | 99.1 |
| Vanillin, ng L^-1^ | 5.00 | 4.94 | 98.8 | 150.0 | 154.4 | 102.9 |

Supplementary Table S3. Intraday (%) and day-to-day (%) precision for aqueous samples at low end (10 ng L^-1^) and high end (100 ng L^-1^) concentrations of the method.

| **Compound** | **Intraday precision, %, low** | **Intraday precision, %, high** | **Day-to-day precision, %, low** | **Day-to-day precision, %, high** |
| --- | --- | --- | --- | --- |
| Acetoin, ng L^-1^ | 2.7 | 2.9 | 3.1 | 3.0 |
| GSM, ng L^-1^ | 1.4 | 1.8 | 1.9 | 2.1 |
| Hexanal, ng L^-1^ | 2.6 | 5.5 | 2.1 | 4.2 |
| Hexanoic acid, ng L^-1^ | 5.8 | 4.3 | 2.9 | 2.0 |
| IBMP, ng L^-1^ | 1.4 | 2.2 | 1.8 | 2.5 |
| IPMP, ng L^-1^ | 3.7 | 1.0 | 1.5 | 1.8 |
| MIB, ng L^-1^ | 2.8 | 3.7 | 1.7 | 2.2 |
| Methional, ng L^-1^ | 2.8 | 3.9 | 1.6 | 2.3 |
| Octanal, ng L^-1^ | 2.3 | 0.8 | 2.1 | 1.9 |
| Octanoic acid, ng L^-1^ | 1.7 | 5.7 | 2.2 | 3.1 |
| Phenylacetaldehyde, ng L^-1^ | 6.3 | 3.4 | 3.1 | 2.9 |
| TCA, ng L^-1^ | 3.6 | 1.0 | 2.6 | 1.7 |
| α-Terpineol, ng L^-1^ | 0.7 | 1.8 | 1.1 | 1.9 |
| Vanillin, ng L^-1^ | 0.6 | 3.7 | 1.5 | 2.1 |

Supplementary Table S4. LODs, LOQs, and linearities (R^2^) of selected off-flavors in aqueous samples analyzed with manual SPME-GC-QQQ.

| **Anion** | **LOD** | **LOQ** | **Linearity, R^2^** |
| --- | --- | --- | --- |
| GSM, ng L^-1^ | 12 | 15 | 0.9676 |
| IBMP, ng L^-1^ | 39 | 42 | 0.9805 |
| IPMP, ng L^-1^ | 5.3 | 9.3 | 0.9336 |
| Methonal, ng L^-1^ | 78 | 163 | 0.9774 |
| MIB, ng L^-1^ | 15 | 16 | 0.9978 |
| Phenylacetaldehyde, ng L^-1^ | 25 | 26 | 0.9955 |
| α-Terpineol, ng L^-1^ | 67 | 89 | 0.9927 |
| Vanillin, ng L^-1^ | 66 | 221 | 0.9900 |

Supplementary Table S5. Measurement data (TAN, NO_2_-N, NO_3_-N, pH, alkalinity, oxygen (mg L^-1^), turbidity, NTU and inlet water flow (m_3_ d^-1^) from Lake Peurunka, in the rearing tank water of the RAS.

| **Date** | **TAN, mg L^-1^** | **NO_2_-N, mg L^-1^** | **NO_3_-N, mg L^-1^** | **pH** | **Alkalinity, mg L^-1^** | **Turbidity, NTU** | **O_2_, mg L^-1^** | **Inlet water, m^3^ d^-1^** |
| --- | --- | --- | --- | --- | --- | --- | --- | --- |
| 1.6. | 1.15 | 0.296 | 70.2 | 7.74 | 122.0 | 9.61 | 6.48 | 3.6 |
| 14.6. | 1.61 | 0.5114 | 69.0 | 7.46 | 94.3 | 15.1 | 8.77 | 3.6 |
| 29.6. | 1.09 | 0.300 | 61.3 | 7.48 | 91.3 | 6.15 | 10.97 | 3.6 |
| 14.7. | 0.66 | 0.139 | 64.4 | 7.70 | 90.7 | 4.95 | 12.78 | 3.6 |

Supplementary Table S6. Average concentrations (ng L^-1^) of the off-flavor compounds in the depuration water after 1, 5, 7, 11, 13, and 15 days.

| **Compound** | **d1** | **d5** | **d7** | **d11** | **d13** | **d15** |
| --- | --- | --- | --- | --- | --- | --- |
| Acetoin, ng L^-1^ | <LOD | <LOD | <LOD | <LOD | <LOD | <LOD |
| GSM, ng L^-1^ | 2.0 | 2.1 | 1.8 | 2.1 | 2.0 | 2.0 |
| Hexanal, ng L^-1^ | 3.0 | <LOD | <LOD | <LOD | <LOD | <LOD |
| Hexanoic acid, ng L^-1^ | 9.9 | <LOD | <LOD | <LOD | <LOD | <LOD |
| IBMP, ng L^-1^ | <LOD | <LOD | <LOD | <LOD | <LOD | <LOD |
| IPMP, ng L^-1^ | <LOD | <LOD | <LOD | <LOD | <LOD | <LOD |
| Methional, ng L^-1^ | 5.6 | <LOD | <LOD | <LOD | <LOD | <LOD |
| MIB, ng L^-1^ | 10.4 | 8.0 | 9.7 | 10.5 | 10.6 | 8.3 |
| Octanal, ng L^-1^ | 15.9 | 12.8 | 14.4 | 16.5 | 17.6 | 15.1 |
| Octanoic acid, ng L^-1^ | <LOD | <LOD | <LOD | <LOD | <LOD | <LOD |
| Phenylacetaldehyde, ng L^-1^ | 1.3 | 0.7 | <LOD | 1.0 | 0.6 | <LOD |
| TCA, ng L^-1^ | <LOD | <LOD | <LOD | <LOD | <LOD | <LOD |
| α-Terpineol, ng L^-1^ | 117 | 52 | 37 | 36 | 55 | <LOD |
| Vanillin, ng L^-1^ | 11.6 | <LOD | <LOD | 11.9 | 10.6 | 6.9 |

Supplementary Table S7. Average concentrations (ng kg^-1^) of the off-flavor compounds in fish muscle detected in the rearing tank and in depuration after 1, 5, 7, 11, 13, and 15 days.

| **Compound** | **Rearing tank** | **d1** | **d5** | **d7** | **d11** | **d13** | **d15** |
| --- | --- | --- | --- | --- | --- | --- | --- |
| Acetoin, ng kg^-1^ | 20.5 | 25.1 | <LOD | <LOD | <LOD | <LOD | <LOD |
| GSM, ng kg^-1^ | 1683 | 1603 | 594 | 516 | 472 | 522 | 454 |
| Hexanal, ng kg^-1^ | <LOD | <LOD | <LOD | <LOD | <LOD | <LOD | <LOD |
| Hexanoic acid, ng kg^-1^ | 51.5 | 20.7 | 28.1 | 46.2 | 47.8 | 26.9 | 16.0 |
| IBMP, ng kg^-1^ | 2282 | 1463 | 1213 | 1129 | 1424 | 954 | 159 |
| IPMP, ng kg^-1^ | 96.6 | 98.7 | 23.8 | <LOD | <LOD | <LOD | <LOD |
| Methional, ng kg^-1^ | 9.9 | 12.3 | 6.5 | 7.2 | 8.0 | 6.5 | 6.8 |
| MIB, ng kg^-1^ | 603 | 408 | 196 | 160 | 186 | 184 | 172 |
| Octanal, ng kg^-1^ | 80.2 | 51.5 | 47.7 | 39.9 | 42.1 | 44.5 | 33.1 |
| Octanoic acid, ng kg^-1^ | <LOD | <LOD | <LOD | <LOD | <LOD | <LOD | <LOD |
| Phenylacetaldehyde, ng kg^-1^ | 110 | 50.2 | 72.2 | 33.5 | 42.4 | 44.5 | 33.1 |
| TCA, ng kg^-1^ | <LOD | <LOD | <LOD | <LOD | <LOD | <LOD | <LOD |
| α-Terpineol, ng kg^-1^ | 29.6 | 11.2 | <LOD | <LOD | <LOD | <LOD | <LOD |
| Vanillin, ng kg^-1^ | 633 | 608 | 540 | 458 | <LOD | 64.5 | <LOD |
